# The NAC transcription factors play core roles in flowering and ripening fundamental to fruit yield and quality

**DOI:** 10.3389/fpls.2023.1095967

**Published:** 2023-02-23

**Authors:** Jianfeng Liu, Yuyuan Qiao, Cui Li, Bingzhu Hou

**Affiliations:** ^1^ Key Laboratory of Plant Molecular Physiology, Institute of Botany, Chinese Academy of Sciences, Beijing, China; ^2^ University of Chinese Academy of Sciences, Beijing, China

**Keywords:** flower, fruit ripening, NAC transcription factor, abscisic acid (ABA), ethylene

## Abstract

Fruits are derived from flowers and play an important role in human food, nutrition, and health. In general, flowers determine the crop yield, and ripening affects the fruit quality. Although transcription factors (TFs) only account for a small part of plant transcriptomes, they control the global gene expression and regulation. The plant-specific NAC (NAM, ATAF, and CUC) TFs constitute a large family evolving concurrently with the transition of both aquatic-to-terrestrial plants and vegetative-to-reproductive growth. Thus, NACs play an important role in fruit yield and quality by determining shoot apical meristem (SAM) inflorescence and controlling ripening. The present review focuses on the various properties of NACs together with their function and regulation in flower formation and fruit ripening. Hitherto, we have a better understanding of the molecular mechanisms of NACs in ripening through abscisic acid (ABA) and ethylene (ETH), but how NACs regulate the expression of the inflorescence formation-related genes is largely unknown. In the future, we should focus on the analysis of NAC redundancy and identify the pivotal regulators of flowering and ripening. NACs are potentially vital manipulation targets for improving fruit quantity and quality.

## Introduction

Transcription factors (TFs) only account for a small quantity of genes in plant transcriptomes (less than 10%), but they control the global gene expression and regulation in plant development and adaptation. The plant-specific NAC (NAM, ATAF, and CUC) TFs constitute a large family, such as 101, 328, 138, and 280 members in tomato, rice, Arabidopsis and tobacco respectively (Liu G. S. et al., 2022). In general, the role of a TF is dependent on its binding to the cis-elements of the target genes, which are associated with nuclear localization, DNA binding, oligomerization, and gene expression. Because the NAC family exists from aquatic green algae to higher terrestrial plants, its members participate in the formation of the organ boundaries of plants transiting from vegetative growth to reproductive growth and are related to flower development and fruit ripening, which are essential to crop yield and quality (Maugarny-Calès et al., 2016; Ma et al., 2017; Mathew and Agarwal, 2018; Forlani et al., 2021; Singh et al., 2021; Liu G. S. et al., 2022). Thereby, in recent years, much progress has been made toward understanding the molecular mechanisms regulated by NAC TFs in flower development (Guo et al., 2022; Liang et al., 2022; Liu X. et al., 2022; Wang et al., 2022; Ghissing et al., 2023) and fruit ripening (Fu B. L. et al., 2021; Gao et al., 2021; Kou et al., 2021; Gao et al., 2022; Li et al., 2022; Liu B. et al., 2022; Qi et al., 2022). In this review, we mainly summarize up-to-date advances on the basic properties, function, and action mechanisms of NACs in flower formation, flowering, and fruit ripening to improve our understanding of the molecular basis of crop yield and quality mediated by NAC TFs.

Specifically, we highlight that the NAC family includes no apical meristem (*NAM*), Arabidopsis transcription activator factors (*ATAF1*/*ATAF2*), and cup-shaped cotyledon (*CUC2*), which all contain a conserved amino acid (aa) sequence at the N-terminal with five subdomains, which not only possess versatile regulatory patterns at the DNA, RNA, and protein levels but also serve as activators or repressors through variable transcriptional regulatory regions at the C-terminal. Plant-specific NACs evolved from the streptophyte green algae to higher plants, concomitant with the transition of aquatic-to-terrestrial living and vegetative-to-reproductive growth, with a line of distinct NACs constituting a complex regulatory network linked to the phytohormones abscisic acid (ABA) and ethylene (ETH) that are essential to flowering and ripening. Thus, NACs are potentially important manipulation targets for improving fruit quantity and quality.

## Basic traits of NAC transcription factors

In response to the aquatic-to-terrestrial environmental niches, the NAC family has shown gradual amplifications in gene size and diversity for the colony of land concurrently with the evolution of additional gene functions for water conductance, cell support, xylem/phloem differentiation, and vasculature formation, adaptive to land living in a sessile manner. Therefore, the present NACs in higher plants have developed a variety of structural and action traits, including DNA binding, nuclear localization, oligomerization/protein–protein interactions, and hierarchical regulatory patterns, using a conserved N-terminal NAC domain for target binding and a diverse C-terminal flexible region for regulating gene expression as activators or repressors.

### NAC domain traits

**Figure 1:**
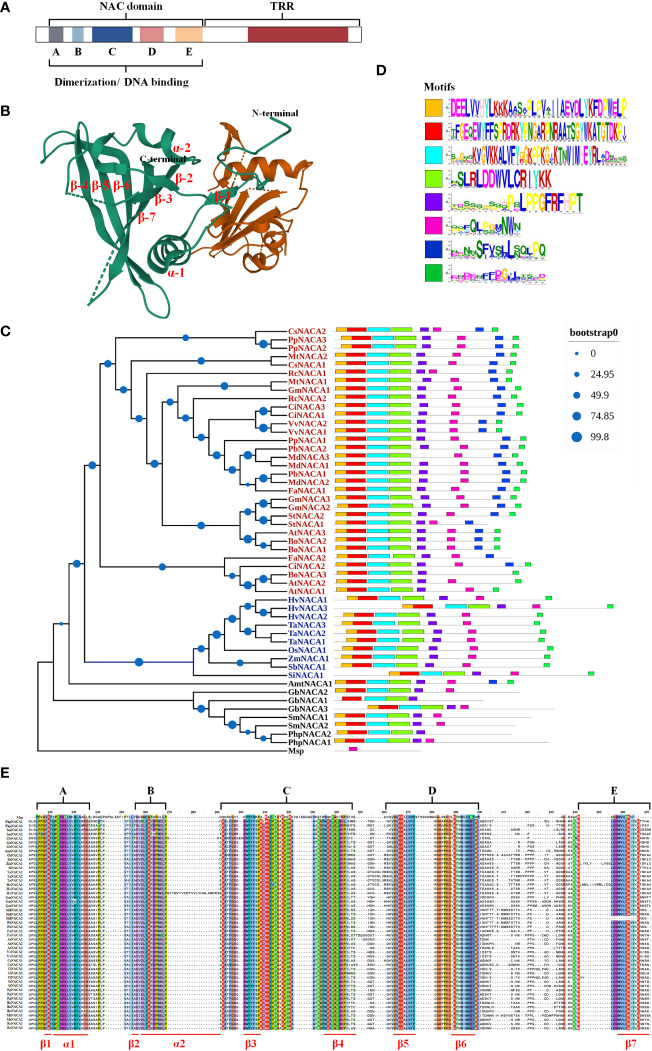
Basic traits of NAC (NAM, ATAF, and CUC) transcription factors (TFs). **(A)** Structure and function of NACs. **(B)** Three-dimensional structure of the Arabidopsis AtNAC019 protein homodimer. The AtNAC019 NAC domain crystal form IV is from the PDB database (PDB DOI: 10.2210/pdb4DUL/pdb). **(C)** Phylogenetic relationship of the NAC TFs. The homologous proteins of the strawberry NAC TF FaNACA2 (FaNAC035) (Martín-Pizarro et al., 2021) were found and verified in 24 species using BLAST and CDD tools, and a phylogenetic tree was constructed with reference to the evolutionary relationship of the NAC TF family in plants. The multiple sequence alignment, sequence pruning, model search, and phylogenetic tree construction were performed in Phylosuite_v1.2.2. The phylogenetic tree was obtained by applying the maximum likelihood method to a matrix of pairwise distances estimated using a JTT (Jones–Taylor–Thornton) model (bootstrap: ultrafast; no. of bootstraps: 10,000). *Blue letters* represent monocots and *red letters* represent dicots. *Msp*, *Mougeotia* sp.; *Php*, *Physcomitrella patens*; *Sm*, *Selaginella moellendorffii*; *Gb*, *Ginkgo biloba*; *Amt*, *Amborella trichopoda*; *Si*, *Setaria italica*; *Sb*, *Sorghum bicolor*; *Zm*, *Zea mays*; *Os*, *Oryza sativa*; *Ta*, *Triticum aestivum*; *Hv*, *Hordeum vulgare*; *At*, *Arabidopsis thaliana*; *Bo*, *Brassica oleracea*; *Ci*, *Citrus sinensis*; *Fa*, *Fragraria ananassa*; *St*, *Solanum tuberosum*; *Gm*, *Glycine max*; *MD*, *Malus domestica*; *Pb*, *Pyrus bretschneideri*; *Pp*, *Prunus persica*; *Vv*, *Vitis vinifera*; *Rc*, *Ricinus communis*; *Mt*, *Medicago truncatula*; *Cs*, *Cucumis sativus*. **(D)** The conserved motifs of all 51 sequences were queried online through the MEME website (https://meme-suite.org/meme/tools/meme). *Motif1* corresponds to the A and B subdomains in the NAC domain, *Motif2* contains the C subdomain, *Motif3* corresponds to the D subdomain, *Motif4* corresponds to the E domain, and *Motif5*–*Motif8* are the conserved motifs in the transcription regulatory region (TRR). **(E)** NAC domain consisting of five subdomains: three highly conserved (A, C, and D) and two diverse (B and E) subdomains.

NAC TFs have the conserved N-terminal NAC region with about 150 aa including both nuclear localization signal (NLS) and DNA binding regions, which are further divided into five subdomains designated from A to E (A, C, and D are more highly conserved compared to B and E) and a C-terminal region as a transcriptional regulatory/activation region (TRR and TAR, respectively) (**Figure 1**). In particular, members of the NAC family have a conserved domain fold containing one β-sheet flanked by an α-helical element, and the C-subdomain has a DNA recognition conserved motif sequence (WKATGT/NDK) that specifically binds to the developmental core CGT(GA) and the stress core CGTG (Mathew and Agarwal, 2018) (**Figures 1B, E**). In addition, NACs include activators and repressors for gene expression and repression, respectively, resulting from the NAC repression domain (NARD) in the D subdomain, such as the rice *NAC020* and *NAC026*, which show bifunctionality having both activation and repression properties (Mathew et al., 2016). In addition, NAC proteins have homodimers and heterodimers, and dimerization is required for stable DNA binding to post-transcriptional and translational modifications in target genes (Chen et al., 2016) (**Figure 1B**). In conclusion, the N-terminal region in NACs contains the repression domain, while the C-terminal region is the transactivation region to a large degree. These properties allow NACs to have multiple regulatory patterns at the transcriptional, post-transcriptional, and translational levels, serving as activators and repressors in response to developmental and environmental cues.

### NAC localization traits

Most of the members of the NAC TF family are localized in the nucleus, although a few of them are situated in other organelles such as the plasma membrane (PM), the cytoplasm, and the endoplasmic reticulum (ER) (Olsen et al., 2005; Kim et al., 2006; Kim et al., 2007; Seo et al., 2008; Wang et al., 2016; Bhattacharjee et al., 2017). Notably, membrane-bound NACs may be anchored in membrane systems, including the PM, ER membrane, and nuclear membrane, in inactive forms. Stimulation by developmental or environmental cues promotes their release and trafficking from the membrane to the nucleus for the control of target gene expression in a common regulatory pattern (Kim et al., 2006; Kim et al., 2007; Seo et al., 2008; Wang et al., 2016; Bhattacharjee et al., 2017). Altogether, the multiple localizations and the translocation traits of NAC TFs implicate various biological functions and regulatory mechanisms in response to developmental and environmental cues.

### NAC evolutionary traits

Environmental factors have played a vital role in the diversification of the NAC family, to a great extent, enabling higher plant survival and prosperity on land. Construction of a phylogeny tree based on the NAC family from green algae to higher plants can help to better understand the origin and expansion of NACs from their aquatic to terrestrial evolution (Mathew and Agarwal, 2018). Higher plant-specific NACs are potentially from the ancestral WRKY protein, concomitant with the transition from an aquatic to a terrestrial environment through two landmark expansions—the evolution of bryophytes from streptophytes, followed by the development of flowering angiosperm plants from vascular plants—which highlight the vital role of NACs in plant evolution (Yamasaki et al., 2013; Maugarny-Calès et al., 2016; Mathew and Agarwal, 2018).

The subfamily of *FaNAC035* (Moyano et al., 2018; Martín-Pizarro et al., 2021), a key regulator of strawberry fruit development and ripening, is a typical case. Phylogenetic analysis showed that this subfamily also followed the evolutionary relationships from aquatic to terrestrial, and some conserved motifs were discovered to be lost when comparing ferns, gymnosperms, and angiosperms (**Figures 1C–E**). We supposed that four subdomains (motif1–motif4) appeared after the first evolutionary expansion; subsequently, the angiosperm NACs evolved to the conserved motif7 and motif8, with motif7 being conserved only in dicotyledons rather than in monocotyledons (**Figures 1C, D**). The number of genes also varies, with monocotyledons having only one member while dicotyledons have more than two members (**Figure 1C**). In brief, the gene numbers and conserved motifs are species-specific and have developed diverse functions in adapting to developmental and stress processes.

Based on wide phylogenetic analysis, the NAC family of green plants is divided into six major groups (groups I–VI) as follows: 1) the basal group I functions in water conduction and cell support; 2) the developmental group II, which includes *CUC1*–*CUC3* (*AtNAC054*, *AtNAC031*, and *AtNAC098*), function in shoot apical meristem (SAM) formation and cotyledon separation, associated with the ETH–auxin pathways; 3) group III has the transmembrane motif (TMM); 4) group IV functions in the timing control of several developmental processes, including flower evolution, such as *AtNAC034* for flowering time, *AtNAC009* for cell division, and *AtNAC042* for longevity; 5) group V regulates the stress responses; and 6) group VI has neo- and/or sub-functionalization in different plant species. In summary, NACs regulate many developmental and stress processes through phytohormones (Ooka et al., 2003; Ernst et al., 2004).

### NAC hierarchical regulatory traits

Higher plants have developed multiple regulatory mechanisms to maintain a balance between the expression and turnover of NACs partly through NAC regulatory loops (Mathew and Agarwal, 2018). In the past years, significant progress has been made toward understanding the regulatory mechanisms of NACs in controlling their homeostasis at the DNA, RNA, and protein levels, including 1) transcriptional regulation by their upstream TFs to form messenger RNA precursors (pre-mRNAs) in the nucleus; 2) post-transcriptional regulation at the nucleotide level, including microRNA (miRNA)-mediated inhibition and/or splicing of pre-mRNAs and their transport from the synthesis site to the action sites; 3) post-translational regulation at the protein level, including the transfer of the cleave-mediated membrane-tethered proteins to the nucleus, which are associated with specific active groups, protein degradation by proteasome machinery, oligomerization, and protein interactions (Mathew and Agarwal, 2018).

Regarding the primary function of NACs in secondary cell wall thickening and xylem vessel element formation, the *NAC secondary wall thickening promoting factor2, NST2/AtNAC066*, and *ORE1* (*ORESARA1/AtNAC092*) are regulated by WRKY12 and EIN2 (ethylene-insensitive 2) (Wild and Krutzfeldt, 2010; Kim et al., 2014), while the secondary wall-associated NAC domain protein1, SND1/NAC012, appears autoregulatory in nature (Wang et al., 2011). In sweet potato (*Ipomoea batatas*), the homeostasis of *IbNAC1* is regulated by the competitive binding of two upstream TFs (the activator bHLH3 and the repressor bHLH4) to its promoter (Chen et al., 2016). Seven vascular-related NAC domains (AtVNDs) can induce the expression of *AtVND7* by binding to its promoter (Endo et al., 2015; Nakano et al., 2015).

NAC TFs have also evolved multiple post-transcriptional regulatory processes, including miRNA-mediated cleavage, alternative splicing, and *trans*-splicing, in regulating the transcripts of NAC functional genes, such as the microRNA164 (miR164)-mediated and organ boundary formation regulatory genes (e.g., *CUC1/AtNAC054, GOBLET/GOB*, and *SlNAM2*) (Samad et al., 2017), the petal size regulatory genes (e.g., *RhNAC100* in *Rosa hybrid*) (Pei et al., 2013), and the cell wall thickening genes (e.g., *PtrWND1B-s* and *PtrWND1B-l* in *Populus trichocarpa*) produced by intron-mediated alternative splicing of the wood-associated NAC TF1B (PtrWND1B) (Zhao et al., 2014).

Nuclear localization is a prerequisite for a protein to function as a TF; thus, NACs contain a bipartite NLS within the D subdomain. However, the Arabidopsis NTL4/AtNAC053 protein accumulates early in the ER, and its C-terminal portion may move to the nucleus after its cleavage from the ER (Kim et al., 2007), as confirmed by several similar reports on NACs targeted to the nucleus by homomerization/heteromerization (Seo et al., 2010; Ng et al., 2013; Lee et al., 2014; Mathew et al., 2016). These findings uncover an important regulatory mechanism of the activity of NACs from early PM/ER localization to nucleus translocation.

In addition, NACs participate in post-translational modifications through reversible acetylation and phosphorylation, protein–protein interactions, and ubiquitin-mediated proteolysis. For instance, the phosphorylation of the membrane-associated NTL6/AtNAC062 by SnRK2.8 (SNF1-related protein kinase) promotes the entry of NTL6 into the nucleus during drought conditions (Robertson, 2004; Kim et al., 2012; Guan et al., 2014). NAC proteins can form both homo- and heterodimers through the conserved β-sheet domain, which is influenced by the TRR/TAR (Zhu et al., 2012). The rice OsNAC29 and OsNAC31 independently interact with OsSLR1 (SLENDER RICE1), a repressor for OsMYB61, which promotes cellulose synthesis by increasing the transcripts of a cellulose synthase gene (Huang et al., 2015). The involvement of NAC1 in auxin signaling is ubiquitinated by SlNAT5, an E3 ubiquitin ligase, which leads to the degradation of NAC1 and the downregulation of the auxin signal in plant cells (Xie et al., 2002; Guo et al., 2015). In summary, NAC TFs have shown varied regulatory mechanisms at the transcriptional, post-transcriptional, translational, and post-translational levels.

## NAC transcription factors regulate the transition from vegetative to reproductive growth and flower formation essential to crop yield

Flowering is a critical agricultural trait associated with fruit yield. Flower initiation and fusion congenitally occur within a single whorl of floral organs to form a connation or union, such as the union of sepals to form a calyx tube, the adnation of stamens and petals to form a corolla tube, and the connation of carpels to form the gynoecium (Ma et al., 2017; Phillips et al., 2020). In most angiosperms, the fusion of various floral organs to form diverse flowers and inflorescences with different floral colors, sizes, shapes, scents, inflorescences, and flowering times that are linked to sepals/petals, stamens, and carpels—from free to fused structures, in addition to a spiral arrangement from the whorled corolla and androecium to a single flower or various inflorescences—is closely related to NAM/CUC3-mediated boundary formation through meristematic development and differentiation (Ma et al., 2017; Phillips et al., 2020).

In the primordium, the formation of distinct boundaries is a critical step to floral organ initiation. The meristematic activity between organs allows floral fusion, which is associated with the early identified genes that are key to boundary formation, separate lateral organ development, and floral fusion, such as the Arabidopsis *LOB* (*lateral organ boundaries*) and *Petunia hybrida NAM/CUC3* (*no apical meristem*), during the transition from vegetative to reproductive growth (Souer et al., 1996; Husbands et al., 2007). Indeed, variations in the expression of the *NAM*/*CUC3* genes correlate with the variations in fusion within floral organs due to *nam* mutants failing to initiate a SAM (Zhong et al., 2016). Similarly, the Arabidopsis genes *CUC1*/*CUC2* and *ATAF1/ATAF2* possess a mutant phenotype similar to the *nam* mutant. These mutations are associated with failure to initiate the SAM and defective to boundary formation, leading to the fusion of cotyledons and floral whorls (Aida et al., 1997). A distinct boundary is required for proper leaflet formation, as evidenced by a tomato *gob* (*goblet*) mutant similar to the *nam* (Berger et al., 2009). Taken together, the tomato *gob* mutant is phenotypically similar to the *nam/cuc3* mutants in both *Arabidopsis thaliana* and *P. hybrida*, in terms of failing to initiate the SAM and the presence of fused cotyledons and floral organs, suggesting a model to meet the requirements of higher *NAM*/*CUC3* expression to increase the separation of the structures and lower expression to promote organ fusion. To a large extent, the NAM/CUC3 family promotes the evolution of floral fusion phenotypes by controlling organ boundary formation (Phillips et al., 2020).

Interestingly, phylogenetic analyses based on comparative genomics across algae and higher plants showed that the NAC family might date back to streptophyte green algae (Maugarny-Calès et al., 2016). From the view of evolution, after the transition of NACs from algae to land organisms, they subsequently expanded throughout land plants, from 20–30 proteins in mosses and lycophytes to over 100 copies in various angiosperm species (Zhu et al., 2012; Pereira-Santana et al., 2015; Maugarny-Calès et al., 2016), among which NAM/CUC3 became a distinct subfamily while the NAM clade (NAM/CUC1/CUC2) was clustered as a sister group of the CUC3 proteins throughout angiosperms (Maugarny et al., 2016). The function of NACs in the formation of vascular tissues promotes flower diversification and makes plants more adaptable to various land environments through seed propagation, which is a landmark event in the evolution of land plants (Maugarny-Calès et al., 2016). In summary, the NAC family plays a pivotal role in the formation of the SAM, which is fundamental to flowering and post-flowering phylogenetic processes, including organ boundary formation, secondary wall thickening, floral development, flowering, embryo and seed development, and fruit development and ripening.

Indeed, the identification of the *nam* mutant and the evolution of the later redundant *CUC1/AtNAC054, CUC2/AtNAC098*, and *CUC3/AtNAC031* uncovered the specialization and diversification of NACs in the formation of embryos and flowers (Souer et al., 1996; Hibara et al., 2006; Raman et al., 2008). At the molecular level, CUC1 directly regulates the expression of *LSH3/LSH4* (*light-dependent short hypocotyls 3/4*) in shoot organ boundary cells, which are associated with the transition from shoot vegetative growth to flower reproductive growth (Takeda et al., 2011). It was further found that SlNAM2 participates in establishing the tomato flower whorl and sepal boundaries (Hendelman et al., 2013). In addition, CUC1–CUC3, NAM, and NH16 can suppress vegetable growth and, thus, promote establishment of the boundaries of the floral organs, such as petals, sepals, and stamens, of plants (Vroemen et al., 2003; Zhong et al., 2016). Therefore, NACs are important in the formation and the maintenance of the different meristem tissues for organ boundary formation and are essential to the transition from vegetative to reproductive growth, which is necessary for embryogenesis, seed formation, and fruit development.

Owing to the vital role of NACs in cell wall biosynthesis in higher plants, it is reasonable that some of the NAC TFs, such as NST1/AtNAC043, NST2/AtNAC066, and NST3/AtNAC012 (NAC secondary wall thickening promoting factors 1–3), positively regulate secondary wall thickening and, thus, function in *Arabidopsis silique* formation and anther dehiscence, which are also negatively regulated by AtNAC053, an anther indehiscence factor (Mitsuda et al., 2005; Shih et al., 2014). In addition, NARS1/NARS2 (NAC-regulated seed morphology1/2), also known as NAC2 and NAM, redundantly mediated Arabidopsis seed embryogenesis by regulating the development and degeneration of ovule integuments (Kunieda et al., 2008). Moreover, the three CUC proteins (CUC1–CUC3) are involved in the initiation and separation of ovules during Arabidopsis reproductive development. CUC1 and CUC2 determine the number of ovules, while CUC2 and CUC3 control organ separation (Gonçalves et al., 2015). Interestingly, NAM-B1 participates in the remobilization of nutrients from the vegetative tissue to grain formation in wheat (Waters et al., 2009). In addition, three rice NAC TFs, namely, OsNAC020, OsNAC026, and OsNAC023, were found to be strongly associated with seed size and grain yield (Mathew et al., 2016), as well as another NAC TF (RhNAC100) in ETH-promoted rose flower opening (Pei et al., 2013). Interestingly, a recent study has found loss of function in tomato OPEN STOMATA 1 (SlOST1), a protein kinase essential for ABA signaling and abiotic stress response. slost1 mutants also exhibited a late flowering phenotype under normal and drought stress conditions through SlOST1 interacting with and phosphorylating the NAC-type TF, VASCULAR PLANT ONE-ZINC FINGER 1 (SlVOZ1), providing insights into a novel strategy to balance drought stress response and flowering (Chong et al., 2022).

**Figure 2:**
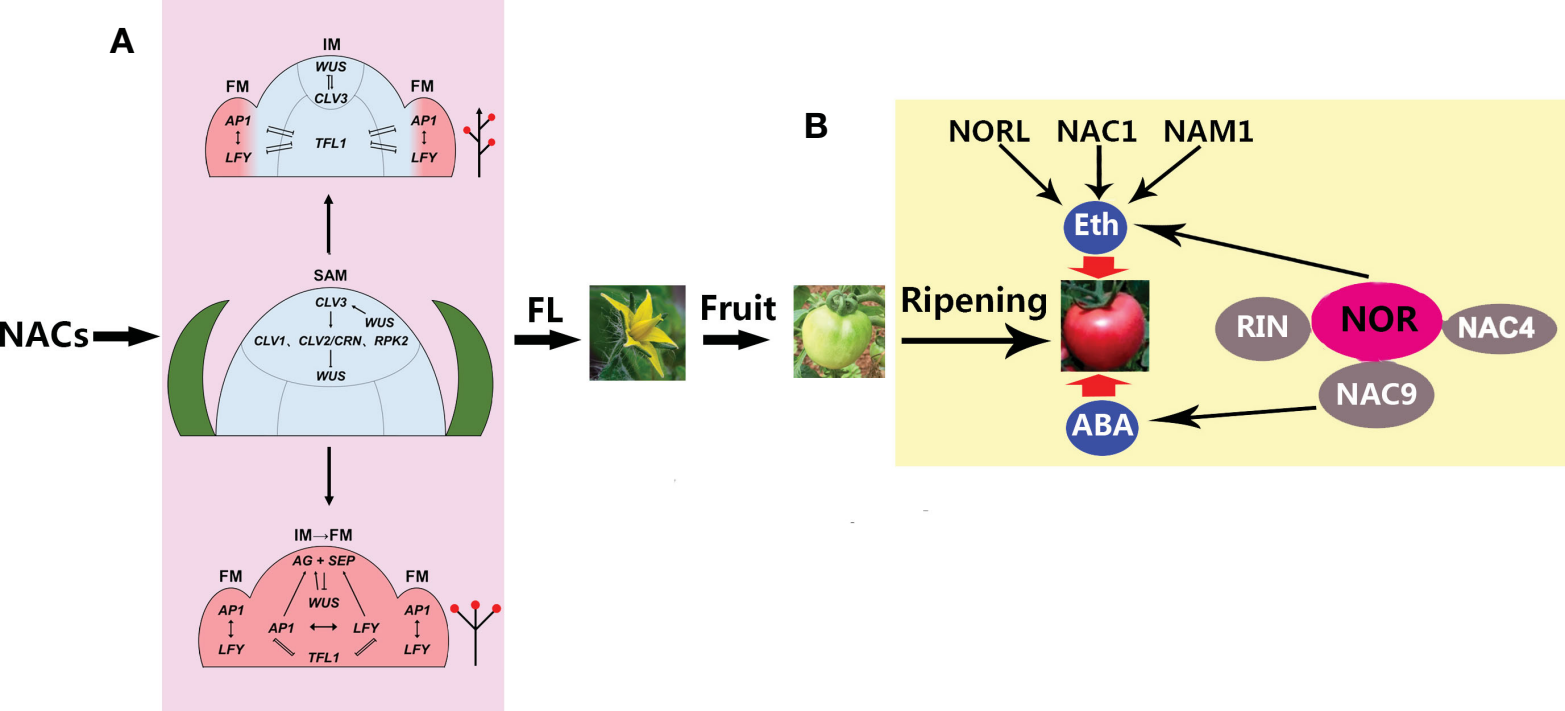
A model for the roles of NACs (NAM, ATAF, and CUC) in flower formation and ripening. **(A)** NACs control the transition of the shoot apical meristem (SAM) to the floral meristem (FM), including indeterminate inflorescence (*up arrow*) and determinate inflorescence (*down arrow*) (Ma et al., 2017). By controlling the relative expression levels of *TFL1* (TERMINAL FLOWER 1) and the FM identity genes, *AP1* and *LFY* (APETALA1 and LEAFY, respectively), in the distal inflorescence meristem (IM), the IM can remain meristematic and generate different numbers of FMs before converting to a terminal flower. A higher relative expression ratio prevents the expression of the FM identity genes *LFY* and *AP1* in the distal IM during the onset of indeterminate inflorescence. However, during determinate inflorescence development, the expression of the FM identity genes in the distal IM can evoke the expression of the C class AGAMOUS (*AG*) and E class SEPALLATA (*SEP*) genes, thus determining the floral fate of the apical meristem. *FL*, flowering. **(B)** NACs control tomato fruit ripening through ethylene (ETH) and abscisic acid (ABA), which are involved in NOR, NORL, RIN, NAM1, and NAC1/4/9. NOR, RIN, and NAC4/9 constitute an interaction complex in which NOR, together with NORL, NAC1, and NAM1, regulates ripening through ETH, while NAC9 regulates ripening through ABA. NOR, non-ripening mutant; NORL, NOR-like; NAM, no apical meristem; RIN ripening inhibitor.

It is worth noting that, in the long-day (LD) model plant Arabidopsis, about 180 regulators have been confirmed to be involved in the control of flowering time, among which CONSTANS (CO) is a central activator, FLOWERING LOCUS C (FLC) is a central suppressor, and SQUAMOSA PROMOTER BINDING LIKE (SPL) is a positive TF, together with their downstream floral TFs, including FT (FLOWERING LOCUS T), SOC1 (SUPPRESSOR OF OVEREXPRESSION OF CO1), and AGL24 (AGAMOUS-like24) that activate the meristemic genes LFY (LEAFY), AP1 (APETALA1), SEP3 (SEPALLATA3), and FUL (FRUITFULL) (Blüemel et al., 2015). A more recent study has reported that strawberries (*Fragaria* sp.), a high-value horticultural crop, initiate flowering during the short photoperiod and low temperatures in the autumn (Liang et al., 2022). With the onset of inflorescence, the SAM may undergo a leaf-to-flower primordium transformation through floral induction, during which SOC1, TFL1, AP1, and CUC2 are vital for floral initiation (Liang et al., 2022). The diversity of NAC proteins is critical for flower formation and fruit development in higher plants. Although much progress has been made toward understanding the molecular mechanisms underlying the formation of a terminal flower through a series of important genes (such as *LFY* and *AP1*) to control the transition from SAM to flower formation (**Figure 2**) (Ma et al., 2017), how NACs regulate the expression of these genes is poorly understood and needs to be urgently explored in the future.

## NAC transcription factors regulate fruit ripening fundamental to quality formation

Fruits include seeds with flower accessories, and fruit ripening is essential to human food, nutrition, and health. Ripening is regulated by plant hormones, including ETH in climacteric fruits and ABA in non-climacteric fruits (Bai et al., 2021; Kou et al., 2021; Li S. et al., 2021). Using the model plant for climacteric fruits, tomato, much progress has been made toward understanding the molecular mechanisms of NACs in fruit ripening through the biosynthesis and activity of ETH and ABA (Bai et al., 2021; Kou et al., 2021; Li S. et al., 2021). As a precursor of ETH biosynthesis, methionine (Met) is transformed into *S*-adenosyl methionine (SAMe) by SAMS (SAMe synthetase), and then SAMe is converted to ETH by 1-aminocyclopropane-1-carboxylic acid synthase (ACS) and 1-aminocylopropane-1-carboxylic acid oxidase (ACO). The ripening signal is relayed through a series of ETH perception and signaling transduction events involving ETH receptors (ETRs), ethylene-insensitive 2 (EIN2), EIN3/EIN3-like, and ETH response factors (ERFs). Similarly, 9-*cis*-epoxycarotenoid dioxygenase (NCED) and ABA 8′-hydroxylase (CYP707A) are key enzymes in ABA homeostasis. The ABA perception and signaling components include PYR/PYL/RCAR receptors, type 2C protein phosphatase (PP2Cs), SNF1-related kinase subfamily 2 (SnRK2), and downstream TFs, such as the ABA-responsive element binding proteins (AREBs) and ABA-responsive element binding factors (ABFs) (Bai et al., 2021; Kou et al., 2021; Li S. et al., 2021).

### Tomato is a model for studying the roles of NACs in fruit ripening

The first landmark finding for NAC function in ripening was from the non-ripening mutant (*nor*) of tomato (*Solanum lycopersicum*) fruit, known as *NAC-SlNOR*, which plays a central role in its ripening (Giovannoni, 2004). However, recent studies have considered that *nor* is a gain-of-function, rather than a loss-of-function, mutant of *SlNOR*, and SlNOR positively regulates fruit ripening by binding to the promoter of *SlACS2* as an activator (Wang et al., 2019; Gao et al., 2020). In addition, *SlNOR* is a direct target gene of SlRIN/SlMADS-RIN (RIPENING INHIBITOR) and SlAREB1 (a TF in ABA signaling). SlRIN and SlAREB1 bind to the *SlNOR* promoter to activate its expression and to promote the expression of *SlACS2/SlACS4* and *SlACO1* for ETH burst, suggesting that the *SlAREB1*-mediated transcriptional regulation of *SlNOR* is involved in the crosstalk between ETH and ABA in ripening (Fujisawa et al., 2013; Mou et al., 2018).

Later, many NAC proteins of *SlNOR* homologs were identified in ripening, such as SlNOR-like1 (SlNAC3/SlNAC4/SlNAC48) that favors ripening by binding to the promoter of multiple target genes involved in both ETH and ABA biosynthesis and signaling, including *SlACS2*, *SlACS4*, *SlACS8*, *SlACO1*, *SlACO6*, *SlCYP707A1*, *SAPK3*, and *SlPYL9*. In particular, SlNAC9 interacts with SlPYL9 and SlAREB1 (Kou et al., 2016; Gao et al., 2018; Yang et al., 2021). In contrast, SlNAC1 (SlNAC033) can directly bind to the promoter regions of *SlACS2* and *SlACO1* and has multiple effects on tomato fruit ripening by inhibiting the biosynthesis of ETH while enhancing the expression of *SlNCED1* and *SlNCED2* for the accumulation of ABA (Ma et al., 2014; Meng et al., 2016). It has been reported that *SlNAC4* may be upstream of ETH biosynthesis by regulating the activities of SlRIN and SlNOR (Zhu M. et al., 2014). On the other hand, the expression of *SlNAC7* was induced by ETH and ABA during color breaking, and, together with *SlNAC6*, it induced the expression of *SlACS2*, *SlACS4, SlACO1*, *SlNCED1*, *SlNCED2*, *SlABA2*, and aldehyde oxidase (*SlAAO1/2*) (Zhu M. K. et al., 2014; Jian et al., 2021). Similarly, a recent report has shown that the new NAC, SlNAM1, is a positive regulator of ripening initiation by promoting the expression of *SlACS2* and *SlACS4* (Gao et al., 2021). In addition, an *Arabidopsis* NAC, JUNGBRUNNEN1 (*AtJUB1*), was overexpressed in tomatoes and resulted in dwarf plants and delayed ripening fruits by directly binding to a line of gene promoters of *GA3ox1* (*GA 3-oxidase 1*) and *DWF4* (*DWARF4*), which are responsible for the biosynthesis of both gibberellin (GA) and brassinosteroid (BR), and indirectly inhibited the expression of *ACS*, *ACO*, *SlERF.H15*, and *SlRIN* (Shahnejat-Bushehri et al., 2017).

Notably, a recent study has found that the NAC-NOR target gene encoding lipoxygenase (SlLOXC) is involved in fatty acid-derived volatile synthesis by epigenetics. NAC-NOR activated the expression of *SlDML2* (DNA demethylase 2) by directly binding to its promoter both *in vitro* and *in vivo* (Gao et al., 2022). On the other hand, the reduced NAC-NOR expression in the *sldml2* mutant was accompanied by the hypermethylation of its promoter, demonstrating a relationship between SlDML2-mediated DNA demethylation and NAC-NOR during tomato fruit ripening (Gao et al., 2022).

In summary, a series of NAC TFs constitute a complex regulation network in tomato fruit ripening, mainly through ETH and ABA homeostasis and signaling levels, confirming that the NAC family plays a central role in fruit ripening.

### NACs play an important role in the ripening of many climacteric fruits

In addition to tomatoes, the NAC family also plays vital roles in many climacteric fruits. For instance, at least 13 NAC genes have been found during apple (*Malus domestica*) fruit ripening (Wang et al., 2011; Zhang et al., 2018; Migicovsky et al., 2021). In banana fruits (*Musa acuminata*), a total of six NACs (*MaNAC1*–*MaNAC6*) were associated with fruit ripening (Shan et al., 2012; Li et al., 2020; Shan et al., 2020; Wei et al., 2021). In pear fruits (*Pyrus pyrifolia*), 185 NACs were found in the genome, among which 1) *PpNAC56* may be related to fruit ripening (Ahmad et al., 2018); 2) *PpNAP1*, *PpNAP4*, and *PpNAP6* may regulate ripening through ETH and ABA; 3) *PpNOR* controls fruit ripening in a conservative manner, similar to *SlNOR*; and 4) PpNAC.A59 directly promotes *PpERF.A16* by binding to its promoter and facilitating the expression of *PpACS1* and *PpACO1*, confirming that it plays an important role in fruit ripening through ETH biosynthesis and signaling (Li et al., 2016; Ahmad et al., 2018; Guo et al., 2021; Tan et al., 2021).

Moreover, during ripening in papaya fruits (*Carica papaya* L.), CpEIN3a could interact with CpNAC2 to activate the biosynthesis of carotenoids, while CpNAC3 could interact with CpMADS4 to activate the expression of *pERF9* and *CpEIL5* (Fu et al., 2017; Fu C. C. et al., 2021). In apricot (*Prunus sibirica*) fruits, 102 NACs were identified in the genome, among which *PsNAC6/13/46/51/41/67/37/59* were highly expressed during the ripening stage (Xu et al., 2021). In kiwifruit (*Actinidia deliciosa*/*Actinidia chinensis*), *AcNAC2*–*AcNAC4* are positive regulators of ripening through ETH production, while *AdNAC2*/*AdNAC3* are promoted by methyl jasmonate (MeJA), suggesting a crosstalk between MeJA and ETH *via* AdNAC2 and AdNAC3 (Wang et al., 2020). In addition, the positive regulators of *AdNAC6* and *AdNAC7* in ripening are degraded by miR164, while this degradation is inhibited by ETH. Interestingly, the miR164–NAC pathway is conserved in both climacteric and non-climacteric fruits, including apple, pear, banana, peach, strawberry, citrus, and grape (Wang et al., 2020; Wu et al., 2020; Nieuwenhuizen et al., 2021).

Notably, a recent study has found that the NAC TF NOR is a master regulator of climacteric fruit ripening. Melon (*Cucumis melo*) (Rios et al., 2017) has climacteric and non-climacteric fruit ripening varieties, such as the climacteric and non-climacteric haplotypes CmNAC-NOR^S,N^ and CmNAC-NOR^A,S^, respectively. A natural mutation in the transcriptional activation domain of CmNAC-NOR^S,N^ contributed to climacteric melon fruit ripening by directly activating its carotenoid, ETH, and ABA biosynthesis genes. In addition, *CmNAC-NOR* knockout in the climacteric-type melon cultivar “BYJH” completely inhibited fruit ripening, while ripening was delayed by 5–8 days in heterozygous *cmnac-nor* mutant fruits. Finally, *CmNAC-NOR* mediated the transcription of the “*CmNAC-NOR*–*CmNAC73*–*CmCWINV2*” module to enhance flesh sweetness. Altogether, the transcriptional activation activity of the climacteric haplotype, CmNAC-NOR^S,N^, on these target genes was significantly higher than that of the non-climacteric haplotype, CmNAC-NOR^A,S^, providing insights into the molecular mechanism of climacteric and non-climacteric fruit ripening in melon (Zhang et al., 2022). In addition, single-cytosine methylome analyses of a DNA demethylase ROS1 (*MELO3C024516*)-knockout mutant revealed changes in DNA methylation in the promoter regions of the key ripening genes, such as *ACS1*, *ETR1*, and *ACO1*, suggesting the importance of DNA demethylation in melon fruit ripening (Giordano et al., 2022). Thus, the NAC family participates in climacteric fruit ripening at multiple regulation levels.

### NACs also play a role in the ripening of many non-climacteric fruits

Strawberry (*Fragaria ananassa*) is a non-climacteric model plant, and 112 *NAC* genes have been found in the genome, including six NACs, *FaNAC006*/*021*/*022*/*035*/*042*/*092*, related to fruit development and ripening. *FaNAC035*/*FaRIF* regulates ripening *via* ABA biosynthesis and signaling, demonstrating the presence of not only a *FaNAC035*-mediated crosstalk among various plant hormones but also a feedback mechanism between the ABA level and its signaling (Moyano et al., 2018; Martín-Pizarro et al., 2021). In addition, *FcNAC1* could participate in ripening regulation by responding to upstream hormone signals, such as those of ABA and auxin (Carrasco-Orellana et al., 2018).

In citrus (*Citrus reticulata*) fruits, an ABA-deficient mutant resulted from the abnormally high expression of *CrNAC036*, which bound to the C*rNCED5* promoter to inhibit its expression. On the other hand, CrMYB68 interacted with CrNAC036 to further inhibit *CrNCED5* expression, finally synergistically retarding fruit ripening (Zhu et al., 2020). Although CitNAC62 is located in the cytoplasm, the nucleus-localized CitWRKY1 may interact with CitNAC62 to promote its movement (Li et al., 2017). In jujube (*Ziziphus jujuba* Mill.) fruits, the NAC *LOC107435239* positively regulated the pericarp lignin accumulation during winter jujube fruit coloration by promoting the transcription of *F5H* (*ferulate 5-hydroxylase*), and *ZjNAC13/14/38/41* showed high expression levels during ripening (Li M. et al., 2021; Zhang Q. et al., 2021). In grape (*Vitis vinifera*) fruits, VvNAC26 interacted with VvMADS9 to induce the gene expression related to ETH and ABA biosynthesis, resulting in early fruit ripening, suggesting that *VvNAC26* promotes ripening by activating the levels of ETH and ABA (Zhang S. et al., 2021). In litchi (*Litchi chinensis*) fruits, *LcNAC13* and *LcR1MYB1* may antagonistically regulate the accumulation of anthocyanin during ripening (Jiang et al., 2019). In watermelon (*Citrullus lanatus*) fruits, *ClNAC68* positively regulated sucrose accumulation during ripening by directly binding to the promoters of both the *invertase* (*ClINV*) and *IAA-amino synthetase* (*ClGH3.6*) to inhibit their expression (Lv et al., 2016; Wang et al., 2021).

In summary, NAC TFs not only directly target genes encoding enzymes related to ripening parameters, softening, sugar, coloring, and aroma but also indirectly affect fruit ripening by regulating the homeostasis of ABA and ETH through conserved and specific mechanisms, which are closely related to fruit quality. ETH-controlled climacteric fruit ripening (such as in tomato) *via* NAC TFs is directly targeted to the ETH biosynthesis-related genes including *ACO* and *ACS*, while also involved in the interaction between ABA and ETH, mostly through ABA to activate *NOR* transcription and ETH synthesis. With regard to non-climacteric fruit ripening, a homolog of the tomato *NOR* in strawberry, the NAC TF FaNAC035/FaRIF, can promote ABA biosynthesis *via FaNCED3*. Thus, NAC TFs play a core role in the two ripening types through the interplay of ETH and ABA.

## Conclusion

The plant-specific NAC TFs not only control the aquatic-to-terrestrial evolution through vasculature formation but also govern the transition from vegetative to reproductive growth through directing SAM differentiation. In addition, they constitute a complex regulatory network that regulates fruit ripening *via* ABA and ETH, improving our understanding of the molecular mechanisms of NACs in fruit ripening (**Figure 2**). Nevertheless, the mechanisms through which NACs regulate the inflorescence formation-related genes are largely unknown. Given that NAC TFs belong to a large family, more focus should be given to the analysis of their redundancy in order to identify pivotal players in the regulation of flowering and ripening. In addition, more attention should be paid to the regulatory network of NACs regulating the ripening of non-climacteric fruits. Finally, NACs are potentially important manipulation targets for improving fruit quantity and quality in the future, likely through water control.

## Author contributions

BH planned the outline of the review. BH and JL collected the available literature and performed re-analyses. BH and JL completed the draft of the paper. YQ and CL compiled and corrected the references. All authors contributed to the article and approved the submitted version.
